# Carbon Nanodots Memristor: An Emerging Candidate toward Artificial Biosynapse and Human Sensory Perception System

**DOI:** 10.1002/advs.202207229

**Published:** 2023-04-18

**Authors:** Cheng Zhang, Mohan Chen, Yelong Pan, Yang Li, Kuaibing Wang, Junwei Yuan, Yanqiu Sun, Qichun Zhang

**Affiliations:** ^1^ Jiangsu Key Laboratory of Micro and Nano Heat Fluid Flow Technology and Energy Application School of Physical Science and Technology Suzhou University of Science and Technology Suzhou Jiangsu 215009 China; ^2^ Department of Materials Science and Engineering Department of Chemistry and Center of Super‐Diamond and Advanced Films (COSDAF) City University of Hong Kong 83 Tat Chee Avenue Hong Kong 999077 China; ^3^ Jiangsu Key Laboratory of Pesticide Sciences Department of Chemistry College of Science Nanjing Agricultural University Nanjing 210095 China; ^4^ School of Chemistry and Life Sciences Suzhou University of Science and Technology Suzhou Jiangsu 215009 China

**Keywords:** artificial synapses, carbon nanodots (CDs), memristors, neuromorphic computing, sensory perception system

## Abstract

In the era of big data and artificial intelligence (AI), advanced data storage and processing technologies are in urgent demand. The innovative neuromorphic algorithm and hardware based on memristor devices hold a promise to break the von Neumann bottleneck. In recent years, carbon nanodots (CDs) have emerged as a new class of nano‐carbon materials, which have attracted widespread attention in the applications of chemical sensors, bioimaging, and memristors. The focus of this review is to summarize the main advances of CDs‐based memristors, and their state‐of‐the‐art applications in artificial synapses, neuromorphic computing, and human sensory perception systems. The first step is to systematically introduce the synthetic methods of CDs and their derivatives, providing instructive guidance to prepare high‐quality CDs with desired properties. Then, the structure–property relationship and resistive switching mechanism of CDs‐based memristors are discussed in depth. The current challenges and prospects of memristor‐based artificial synapses and neuromorphic computing are also presented. Moreover, this review outlines some promising application scenarios of CDs‐based memristors, including neuromorphic sensors and vision, low‐energy quantum computation, and human–machine collaboration.

## Graphical abstract

In this review, the main advances on CDs‐based memristors and their state‐of‐the‐art applications in artificial synapse, neuromorphic computing, and human sensory perception systems are summarized. The structure–property relationship and resistive switching mechanism of CDs are discussed in depth. This work provides instructive guidance to prepare high‐quality CDs and gives some promising application scenarios for memristors‐based neuromorphic sensor and vision.

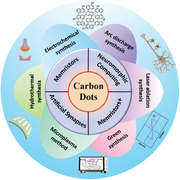

## 1 Introduction

Since the information industry has grown rapidly over the past years, developing high‐performance data storage and computing systems is becoming urgent.^[^ ^1^ ^]^ The traditional solution is to scale down electronic components and increase the density of device cells per unit area. Nevertheless, this strategy is limited by Von Neumann bottleneck and technological challenges in design, manufacturing, and energy dissipation. To address these issues, researchers are devoting substantial efforts to exploring a new generation of ultrahigh‐density non‐volatile memory (NVM) technology and algorithm.^[^ ^2^ ^]^ Among the current burgeoning techniques, two‐terminal memristors with a metal/insulator/metal (MIM) structure have emerged as promising information storage and processing technology, due to their excellent characteristics of low power consumption, high‐frequency and high‐density data programming, and reliable compatibility with complementary metal‐oxide‐semiconductor (CMOS) circuits for multi‐integration applications.^[^ ^3^ ^]^

Recent studies have demonstrated that memristors with MIM structures can be used to construct efficient in‐memory computing systems with enhanced computation capability.^[^ ^4^ ^]^ Unlike conventional computers with separated memory and processing units, the two‐terminal memristor with integrated storage and computing architectures is capable of overcoming the von Neumann bottleneck. Further cutting‐edge research is to develop brain‐like bionic electronic devices that adopt memristors as equivalent electronic components. Considering that the human brain can perform the tasks of memory, learning, computation, cognition, and emotions, a biomimetic neuromorphic network comprising trillions of neurons connected by more than 10^15^ synapses is anticipated to be established via memristor technology. In the long run, exploiting a memristor device array to simulate synaptic connections in a biomimetic neuromorphic brain or human sensory perception system will become a revolutionary technology.^[^ ^1c^ ^]^

During the past decade, a large number of studies have been conducted on the preparation methods of memristive materials, tunable performance, and device manufacturing. To date, a wide range of inorganic and organic memristive materials have been intensively studied, including inorganic oxides,^[^ ^5^ ^]^ phase‐change materials,^[^ ^6^ ^]^ organic small molecules,^[^ ^4^, ^7^ ^]^ polymers,^[^ ^8^ ^]^ organic nanocrystals,^[^ ^9^ ^]^ semiconductor quantum dots (SQDs),^[^ ^10^ ^]^ van der Waals and 2D materials,^[^ ^11^ ^]^ novel carbon materials,^[^ ^12^ ^]^ biomaterials,^[^ ^13^ ^]^ MXenes,^[^ ^14^ ^]^ ferroelectric materials,^[^ ^15^ ^]^ perovskites,^[^ ^16^ ^]^ as well as their hybrid composites.^[^ ^17^ ^]^ For organic memristive materials, one of the challenges is their insufficient stability and low tolerance to ambient conditions, whereas inorganic memristive materials are limited by their poor flexibility and tunability. By contrast, the emerging solution‐processable carbon nanodots (CDs) exhibit unique physicochemical and photochemical properties, such as excellent charge storage capability, tunable energy level, and photo‐/electro‐luminescence, offering an appealing path to achieve high‐performance photosensitive logic sensors and electronic devices.^[^ ^18^ ^]^ More importantly, 0D CDs possess attractive low toxicity, environmental friendliness, biocompatibility, and simple synthesis, which have shown wide applications in the field of biosensors, chemical sensors, nanomedicine, bioimaging, solid‐state batteries, photocatalysis, electrocatalysis, and so on.^[^ ^19^ ^]^ Particularly, CDs‐based memristors have emerged as promising candidates to implement artificial synapses and human sensory perception systems, due to their outstanding structural stability and quantum confinement effects.

**Figure 1:**
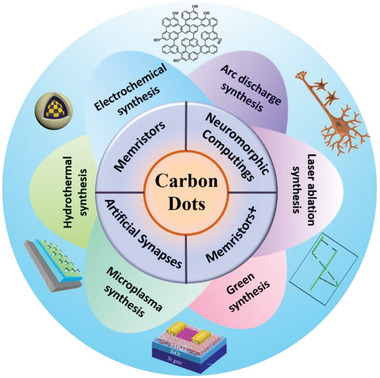
Overview diagram of CDs, involving synthesis method, material design, the memristive, synaptic, neuromorphic computing applications, and possible application scenario (memristor+). Image at the bottom: Reproduced with permission.^[^ ^55^ ^]^ Copyright 2016, Royal Society of Chemistry. Image at the top left: Reproduced with permission.^[^ ^2b^ ^]^ Copyright 2022, Wiley.

In this contribution, we focus on the recent progress of CDs‐based memristors for artificial synapses and neuromorphic computing applications. This review begins with a brief introduction of CDs materials, involving general chemical makeup and basic physical properties. The various synthetic approaches for CDs are summarized. Then, we present the strategies to construct CDs‐related composite materials. Finally, the memristive, synaptic, and neuromorphic computing performances of CDs‐based memristors, as well as their intrinsic mechanisms, are well discussed. It is believed that this article will offer a comprehensive overview on current achievements and challenges of CDs‐based memristors (**Figure** **1**), and open new perspectives toward the future memristor development in the post‐Moore era.

## 2 Basic Profile of CDs Materials

Since Xu et al. discovered the CDs during the separation and purification of single‐walled carbon nanotubes (SWCNTs) in 2004, more and more investigations have proceeded to develop variable CDs materials and their physicochemical characteristics. Typically, CDs are quasi‐spherical nanoparticles, composed of amorphous or nanocrystalline cores with the sources of graphitic or turbostratic carbon (sp^2^ carbon) or fused with sp^3^ hybridized carbon insertions. Most 0D CDs materials normally possess 10^3^ to 10^6^ atoms with an average size of 1–10 nm. There are considerable amounts of oxygen‐containing groups existing on their surfaces. The oxygen content in CDs varies from 5% to 50% (in weight), which relies on different synthetic conditions. It is worth noting that the oxygen content largely affects the physicochemical properties, such as fluorescence and solubility in aqueous and non‐aqueous solvents. More importantly, the suitable chemically‐reactive groups at the CDs surface endow more possibilities for surface passivation and functionalization with various polymeric, organic, biological, or inorganic materials. Compared with conventional SQDs, CDs have unique attributes of easy synthesis, benign chemical composition, and tunable physicochemical and photochemical properties. However, some issues of complex purification and functionalization procedures, ambiguous geometry, composition and structure, must be tackled as soon as possible. Till now, several reviews on CDs, concerning different aspects of synthetic methods, surface functionalization, growth mechanisms, physicochemical properties, and technical applications, have been published, which help us to better understand the structural and chemical details of CDs.^[^ ^20^ ^]^

## 3 Preparation of CDs Materials

**Figure 2:**
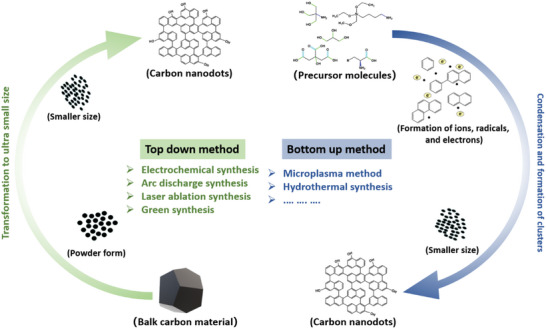
The typical synthetic methods of CDs, which have been classified into the top‐down method (left) and bottom‐up method (right).

In this section, the preparation methods of CDs are generally classified into top‐down and bottom‐up categories. As illustrated in **Figure** **2**, the top‐down method can allow us to prepare small‐size CDs through the destruction or dispersion of macromolecules (e.g., carbon nanotubes, carbon soot, activated carbon, nanodiamonds, graphite, and graphite oxide (GO)), while the bottom‐up method involves chemical polymerization and carbonization of small molecules (e.g., citrate, polymer‐silica nanocomposites, and carbohydrates). The bottom‐up method requires harsher reaction conditions (e.g., high pressure, high temperature, and strong oxidative agents) to convert sp^3^ carbon into sp^2^. The top‐down synthetic routes primarily involve electrochemical oxidation synthesis, arc discharge synthesis, laser ablation synthesis, green synthesis, etc. The specific bottom‐up methods are microplasma synthesis, hydrothermal treatment synthesis, etc. To better comprehend these synthetic processes, we have concisely described them in the following content and concluded their pros and cons in **Table** **1**.

**Table 1:** The advantages and disadvantages of different synthetic methods for CDs

Methods	Precursors	Advantages	Disadvantages	Ref.
Electrochemical synthesis	Graphite rod	High purity, adjustable size, large scale, high productivity	Ease of aggregation	[21]
Arc discharge synthesis	Nano‐carbon material	Good water solubility	Uncontrollable size, low purity	[25]
Laser ablation synthesis	Carbon material	Good water solubility, adjustable size	Complex and immature process, high cost	[29]
Green synthesis	Walnut shell	Simple fabrication, low‐cost	Uncontrollable size	[32]
Hydrothermal synthesis	Citric acid, glucose	High yield, Eco‐friendly, low‐cost, high efficiency, no toxicity, diverse chemical property	Low yield, uncontrollable size	[35]
Microplasma synthesis	Hydrocarbon	Simple fabrication, no toxicity	Complex apparatus, expensive precursors	[43]

### 3.1 Electrochemical Synthesis

**Figure 3:**
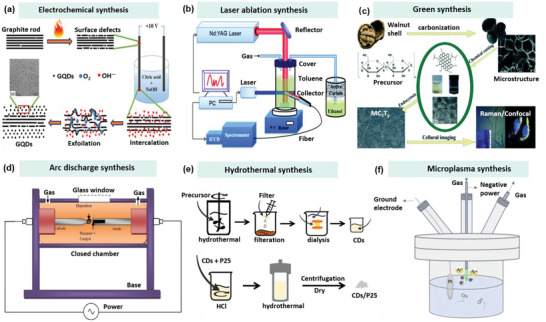
The schematic diagram of different preparation methods of CDs: a) Electrochemical synthesis. Reproduced with permission.^[^ ^21^ ^]^ Copyright 2012, Royal Society of Chemistry. b) Laser ablation synthesis. Reproduced with permission.^[^ ^25^ ^]^ Copyright 2010, American Chemical Society. c) Green synthesis. Reproduced with permission.^[^ ^29^ ^]^ Copyright 2014, Elsevier. d) Arc discharge synthesis. Reproduced with permission.^[^ ^32^ ^]^ Copyright 2019, American Chemical Society. e) Hydrothermal synthesis. Reproduced with permission.^[^ ^35^ ^]^ Copyright 2018, Wiley. f) Microplasma synthesis. Reproduced with permission.^[^ ^43^ ^]^ Copyright 2020, Elsevier.

Electrochemical synthesis has been considered as an efficient method to prepare CDs (**Figure** **3**a). In a recent study, Ming et al. synthesized high‐quality CDs with an average size of 3–6 nm in a large scale through a simple electrochemical oxidation approach. First, a graphite rod was inserted into ultrapure water (600 mL) as the anode (18.4 MΩ cm^−1^), parallel to the second graphite rod as the opposite electrode with a distance of 7.5 cm. Then, a static potential of 15–60 V was applied to both electrodes supplied by a direct current (DC) power source. During continuous stirring for 120 h, the anode graphite rod was slowly corroded in the reactor to form a dark yellow solution (comprising CDs and large‐scale GO). To remove the precipitated GO and graphite particles, the mixed solution was filtered through a slow quantitative filter paper, followed by centrifuging for 30 min at 2.2 × 10^4^ rpm. Finally, the water‐soluble CDs were obtained with a yield of 16.5%.^[^ ^21^ ^]^ Notably, the as‐prepared CDs exhibited high crystallization, excellent water dispersion, and high purity, which can be directly used without further purification. In another recent study, Li et al. reported a one‐step electrochemical treatment technology to prepare CDs by implementing the oxidation reaction of sodium hydroxide and ethanol. As a result of its convenience, low energy consumption, and environmental friendliness, this modified electrochemical method is suitable for large‐scale production of fluorescent nano‐carbon materials.^[^ ^22^ ^]^ Overall, the electrochemical approach of synthesizing CDs is a facile method in an economical and environment‐friendly way, which displays the advantages of mass production, high productivity, and high purity. In comparison with other synthetic routes, most CDs obtained via electrochemical synthesis display distinct multi‐layer nanostructures.^[^ ^23^ ^]^ Additionally, CDs can be easily tailored into different sizes during the synthetic process, which can be applied in different scenarios.^[^ ^24^ ^]^

### 3.2 Laser Ablation Synthesis

During the laser ablation synthesis, a high‐energy laser pulse illuminates the target surface to create a thermodynamic state with high pressure and temperature (Figure 3b), followed by rapidly heating and evaporating a plasma state. As a result, the carbon vapor will be crystallized to generate the target nanoparticles.^[^ ^25^ ^]^ Li et al. developed a family of CDs materials using a laser‐ablation synthesis method. By passivating nanoparticles in organic chemical solvents, visible, stable, and adjustable photoluminescence (PL) characteristics could be achieved. In this work, nano‐carbon materials were used as the starting materials, while a single solvent was added as the liquid medium. The raw precursor owns turbostratic structures with an average size below 50 nm and shows prominent PL properties. After laser irradiation, the PL of nano‐carbon suspension would be changed, due to the transformation of nano‐carbon materials into a nuclear shell structure with an amorphous outer layer and polygon or onion carbon inside, which was confirmed by high‐resolution transmission electron microscope (HRTEM). The laser irradiation passivation played an important role in modifying PL performance, as revealed by X‐ray photoelectron spectroscopy (XPS) measurements.^[^ ^26^ ^]^ In the work of preparing CDs through laser irradiation by Yu et al., anhydrous toluene was chosen as the carbon source, and a laser furnace was used to regulate the CDs sizes.^[^ ^27^ ^]^ As a result, it produced water‐soluble CDs with narrow size distributions. Furthermore, the quantum size of CDs can be well controlled by adjusting technical parameters, such as the irradiation time and laser intensity.^[^ ^26^, ^28^ ^]^ In short, laser ablation synthesis has been considered an effective way of preparing CDs, however, the complicated apparatus and operation procedures make it very costly and difficult for mass production.

### 3.3 Arc Discharge Synthesis

Arc discharge synthesis is a method of reconstituting the decomposed carbon atoms of bulk carbon precursors around a cathode electrode, driven by a gas plasma in a sealed reactor (Figure 3c). Specifically, it is possible to produce a high‐energy gas plasma through the electrodes in the reactor, where a high temperature of 4000–6000 K under the constant current effect is generated. The decomposed carbon vapor then aggregates and assembles around the cathode to create CDs products.^[^ ^29^ ^]^ In 2004, Xue et al. produced SWCNTs by arc discharge synthesis. Three kinds of carbon nanoparticles with different formula weights and fluorescence properties were simultaneously discovered.^[^ ^30^ ^]^ Soon afterward, these three carbon nanoparticles were finally proven as CDs, which is believed to be the first report of CDs. The as‐prepared CDs exhibited excellent water solubility but a wide distribution of particle sizes due to their variety of sizes. In view of the large‐size CDs with a reduced specific surface area, there are some constraints to their practical applications. Moreover, many tiny impurities may be introduced into CDs during the synthetic process.^[^ ^31^ ^]^ Till now, arc discharge synthesis technology is not yet mature enough, which deserves a great deal of efforts to fully understand the influencing factors during the synthesis and assembly of CDs, such as the arc temperature, electrode geometry, power supply type, current intensity, and other correlation parameters.

### 3.4 Green Synthesis

Green synthesis means that CDs can be manufactured from a wide range of biomass wastes, such as food waste (e.g., lemon, orange, and watermelon peel, fennel seeds, coffee grounds, and orange juice), agricultural waste, and so on (Figure 3d).^[^ ^32^ ^]^ It has been demonstrated by Cheng et al. that walnut shells composed of natural cellulose can be utilized to prepare CDs. The synthetic route involves carbonization and acid treatments, finally producing CDs with an average size of 3.4 nm.^[^ ^33^ ^]^ TEM analysis revealed the existence of zigzag and armchair edges on CDs. According to the calculations, the lattice spacing distance of CDs was 0.258 nm, which was in accordance with the (100) crystal planes in graphitic carbon. Under a light irradiation by 360–460 nm, photoluminescence CDs emitted green fluorescence, which could be influenced by their shape, size, and edge‐state effect. Furthermore, the hydrophilic CDs materials exhibited pH‐sensitive characteristics and up‐converted photoluminescence, which can be further used to detect chemical compounds, biomolecules, and image cells. More importantly, green synthesis has no requirement of expensive materials, redundant post‐treatment or complex experimental equipment, demonstrating great value in economy and industrialization.^[^ ^34^ ^]^

### 3.5 Hydrothermal Synthesis

Hydrothermal synthesis method is one of the widely‐used bottom‐up approaches for CDs fabrication (Figure 3e). Basically, an organic precursor solution is placed in an autoclave to produce CDs under high temperatures.^[^ ^35^ ^]^ This technique has the advantages of high yield (up to 80%), low cost, high efficiency, no toxicity, and no pollution. Using hydrothermal approach, Kuo et al. synthesized hydrophobic and hydrophilic graphene quantum dots (GQDs) by exfoliating and dissolving graphite flakes. These GQDs were successfully applied to the supercapacitor, inkjet printing, and non‐volatile bio‐memristor industries.^[^ ^36^ ^]^ The device of indium tin oxide (ITO)/GQDs‐albumen/Al exhibited high ON/OFF current ratios (>10^4^) and stable switching endurance over 250 cycles. The retention stability of the high resistance state (HRS) and high resistance state (LRS) state can be maintained for over 10^4^ s. As reported, a variety of precursors have been reported to produce CDs, such as glucose,^[^ ^37^ ^]^ sucrose,^[^ ^38^ ^]^ citrate,^[^ ^39^ ^]^ chitosan,^[^ ^40^ ^]^ orange juice,^[^ ^41^ ^]^ and so on. In 2019, Yro et al. successfully fabricated CDs through the hydrothermal synthesis method, where the biowaste carbon sources (such as calamansi peels and pineapple crowns) were used.^[^ ^42^ ^]^ Notably, the precursors tend to dominate both the structural and chemical properties of CDs, and possible applications. Therefore, it is important to deliberate on the structural properties of organic precursors, such as the number of carbon atoms, carbon chain length, functional groups, etc. However, hydrothermal synthesis of CDs is limited by the uncontrollable nanosize, and the low yield may occur due to the loss over the reactor walls.^[^ ^23^, ^32^ ^]^

### 3.6 Microplasma Synthesis

Microplasma technology has been widely utilized to prepare diverse nanomaterials (e.g., carbon nanotube (CNT), CDs),^[^ ^43^ ^]^ in which a microplasma discharge is used to activate the reactive precursor.^[^ ^44^, ^45^ ^]^ Generally, phase‐arc plasma synthesis mainly involves the decomposition of hydrocarbons in microplasma reactors. Here, we introduce microplasma synthesis (one of the well‐known examples). Ma et al. could prepare fluorescent CDs in pure isopropanol solvent through microplasma synthesis (Figure 3f), involving a convenient, efficient, and environmentally friendly process without toxic chemicals and complex purification procedures.^[^ ^46^ ^]^ Since the microplasma synthesis requires low pressures, expensive equipment, and expensive precursors compared with other synthetic methods, this method shows some obvious disadvantages, such as high costs, low yields, and difficult mass production. Particularly, the nucleation and growth of particles, and the CDs shapes, can be affected by controlling the short residence time during reactions. Therefore, it is facile to generate high‐quality CDs with a narrow size distribution. Furthermore, the microscale reactor can realize high‐pressure emissions, ensuring relative safety during preparation.

## 4 Construction of CDs‐Related Composite Materials

In general, it is not an appropriate choice to utilize pure CDs as the active layer of the memristor and artificial synaptic device, due to their poor dispersion and ease of aggregation. One of the solutions is to construct CDs‐based composites or heterojunction materials. Some emerging 2D organic/inorganic materials, polymeric materials, and biomaterials have been recognized as promising platforms, where these materials could be polymethyl methacrylate (PMMA), polyvinylpyrrolidone (PVP), silk cellulose, GO, MXene, h‐BN, covalent organic framework (COF), and metal–organic framework (MOF).^[^ ^47^ ^]^ According to some previous reports, it has been known that CDs can serve as strong charge traps, which may change the ways of charge transfer in CDs‐related composite materials or induce the growth and fracture of conductive filaments (CFs) in memristors.^[^ ^48^ ^]^ Several typical cases have been introduced below to illustrate the construction strategies for CDs‐based composite materials.

**Figure 4:**
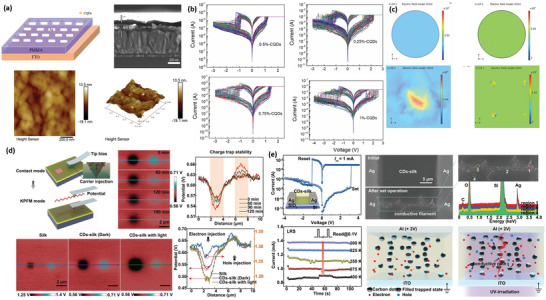
a) Schematic structure and SEM profiles of the Ag/PMMA@CDs/FTO device. b) *I*–*V* curves of PMMA@CDs with different doping ratios of CDs. c) The COMSOL simulation of the 10th‐second variation images under 1 V voltage bias. Reproduced with permission.^[^ ^49^ ^]^ Copyright 2021, American Institute of Physics. d) KPFM measurement of CDs‐silk film to prove the charge trapping capability. e) Experimental SEM images of the CDs‐silk‐based device and schematic diagrams describing the formation of a conductive path. Reproduced with permission.^[^ ^35^ ^]^ Copyright 2018, Wiley.

For example, Li et al. embedded CDs in PMMA to fabricate a sandwich‐structured Ag/PMMA@CDs/FTO resistive random‐access memory device (RRAM).^[^ ^49^ ^]^ By integrating CDs with dielectric PMMA films, the homogeneity and stability of the resistive switching behaviors have been improved, which is far superior to those of the PMMA‐based RRAMs without inserted CDs. Intriguingly, the resistive switching characteristics can be easily tuned by adjusting different doping ratios of CDs in PMMA matrix. As shown in **Figure** **4**a, non‐volatile memory behaviors were obtained in all devices. Comparing with the five different doping concentrations (0%, 0.25%, 0.5%, 0.75%, and 1% volume ratio) of CDs in PMMA matrix, the PMMA@0.5%CDs‐based RRAM exhibited the lowest set/reset voltage and the most homogeneous threshold distribution, indicating that 0.5% CDs in PMMA is the best doping concentration for the CDs‐based composite materials. The Ag/PMMA@0.5%CDs/FTO device also demonstrated an acceptable memory window and stable endurance to ambient conditions. Based on the experimental results and theoretical simulation by COMSOL multiphysics software (Figure 4b), the CDs insertions in PMMA could serve as trapping reservoirs, which received the carriers injected from metal electrodes under the applied voltage. As a result, the PMMA@CDs composite film could enhance the local electric field and further induce ordered growth and fracture of CFs.

Ali et al. fabricated a composite of GQDs and PVP on a flexible polyethylene terephthalate (PET) substrate by electro‐hydrodynamic technique.^[^ ^50^ ^]^ A 3 × 3 crossbar device array was fabricated and exhibited superior performances of low threshold voltage (±1.8 V), high OFF/ON ratio (10^14^), long‐term stability (>30 days), excellent bendability (10^3^ cycles), stable switching endurance over 500 cycles, and high potential for flexible and wearable electronic applications. Ivanov et al. incorporated GQDs into fluorinated graphene (PFG), which acted as the active layer in Ag/PFG/PVA(polyvinyl alcohol)/Ag memristor crossbar array. The switching memory behaviors could be well maintained over a whole year, even under a wide temperature range of 80–300 K. They attributed these resistive memory behaviors to the active traps at the PFG/PVA interface, where the activation trap energies were as low as 0.05 eV and the time for fast charge‐carrier emission from the localized states was 5 µs.^[^ ^51^ ^]^

Lv et al. reported a case of RRAM composed of photonic CDs and silk cellulose. It was demonstrated that CDs exhibited light‐tunable charge trapping ability, which could play an imperative role in photosensitive RRAM. Figure 4d presented the images of the contact potential difference when a voltage was applied between the conductive tip and CDs@silk layer. In contrast to pristine silk‐based films, the CDs@silk layer yielded an increased surface potential induced by electron injection and negligible variations, which was confirmed by Kelvin Probe Force Microscopy (KPFM) measurements. Besides, CDs‐based composite offered excellent biodegradability and biocompatibility, facilitating the photo‐regulation of bioelectronic storage or implantation devices.^[^ ^52^ ^]^ For the Ag/silk@CDs/ITO device, the phototunable memory behavior was primarily determined by the formation/rupture of conductive Ag filament (Figure 4e). The photogenerated electrons at the CDs and silk interface can be captured by CDs, acting as a local gate. These previous studies have indicated that introducing CDs into composite matrixes can optimize the uniformity of switching voltage distribution and improve the stability of resistive switching performance, which provides a potential condition for biomimetic synapse devices and neural component applications in the future. The following content will discuss and summarize the intrinsic memristive mechanism in depth.

## 5 CDs‐Based Memristors

**Figure 5:**
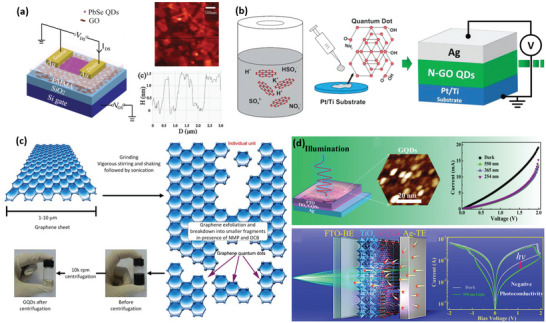
a) Schematic diagram of the ambipolar GDs‐based FET devices. Reproduced with permission.^[^ ^55^ ^]^ Copyright 2016, Royal Society of Chemistry. b) Schematic diagram of the two‐terminal BT devices. Reproduced with permission.^[^ ^54^ ^]^ Copyright 2019, Wiley. c) Schematic of GQDs synthesis by the co‐solvent, mechanical grinding, and ultra‐sonication‐assisted method. Reproduced with permission.^[^ ^59^ ^]^ Copyright 2016, Royal Society of Chemistry. d) Schematic diagram and the performance of the memristor with a structure of Ag/GQDs/TiO*_x_*/FTO under illumination. Reproduced with permission.^[^ ^62^ ^]^ Copyright 2021, Wiley.

Since the memristor concept was proposed by Chua in 1971 and further implemented by HP laboratory in 2008,^[^ ^53^ ^]^ a wave of researches on memristors has been triggered both in academia and industry. To satisfy practical requirements, the high‐performance memristor devices should have fast switching speed (<1 ns) and low energy consumption (<10 fJ). Two typical device structures of the field‐effect transistor (FET) and two‐terminal bottom‐top (BT) devices are illustrated in **Figure** **5**a,b.^[^ ^54^ ^]^ By comparison, FET‐type memristors can integrate with CMOS circuits and readily achieve bipolarity memory properties.^[^ ^55^ ^]^ However, the two‐terminal BT memristors have the advantages of easy fabrication, low cost, and so on. Both organic channel FET and BT devices have gained great achievements over the past years. In 2018, Raeber et al. reported all‐carbon digital‐type resistive behavior (D‐RS) based on amorphous carbon materials, manifesting a great potential of advanced carbon materials in flexible electronics, especially memristors.^[^ ^56^ ^]^ Recently, Pei et al. demonstrated a CDs‐based BT memristor model that was triggered by carbon conducting filaments (CCFs).^[^ ^9^ ^]^ Compared to metal CFs, CDs show a less active characteristic and reduced randomness of diffusivity in the functional layer under a voltage bias. Thus, the growth and nucleation of CCFs can be easily manipulated, which is beneficial to upgrading the memristive performance, including lower turn‐on voltages and more concentrated switching voltage distribution.^[^ ^57^ ^]^ Some reports have contributed the unsatisfactory stability to the random ion transport or diffusion, and the formation and fracture of disordered dendritic filaments, which become an obstacle on the way from labs to practical applications. Thus, it is worthy of exploring effective strategies to control RRAM behaviors and achieve high‐quality memristors. These results strongly confirmed the significance of CCFs in modulating the uniformity and stability of memory performance.^[^ ^58^ ^]^

### 5.1 CDs on Organic Substances

Although a variety of memristive materials have been developed, there still remain some difficulties in poor flexibility and biocompatibility. By contrast, nanoscale CDs stand out from these conventional organic/inorganic memristive materials, manifesting rich application prospects in flexible and biocompatible electronic devices.^[^ ^19b,e^ ^]^ GQDs, as a representative of CDs, have received adequate investigations in the field of memristors.^[^ ^59^ ^]^ Figure 5c shows the schematic diagram of preparation procedures of GQDs, involving co‐solvent synthesis, mechanical grinding, and ultra‐sonication. The as‐obtained GQDs were then attached onto the surface of GO substrate via a solution‐processable method, serving as charge‐trapping centers. An ITO/GO@GQDs(0.5 wt%)/Ni device was fabricated and showed a tristable switching resistance, named as OFF, ON1, and ON2 states, which can be employed in multilevel data‐storage applications.^[^ ^60^ ^]^ The ternary memory device, operating at the SET1/SET2 and RESET voltage of −0.9/−1.7 V and 5.15 V, displayed a high ON2/ON1/OFF current ratio (10^3^:10^2^:1), a long retention time (10^4^ s) and 100 endurance cycles. According to an in‐depth study, the ternary memory behavior was attributed to the charge trapping/detrapping mechanism due to the incorporation of GQDs into GO layer. Wang et al. combined multi‐walled carbon nanotubes (MWCNTs) with GQDs to fabricate Al/MWCNT@GQDs/ITO memristor.^[^ ^23^ ^]^ Due to the high UV sensitivity and tunable resistance property of MWCNT@GQDs, the memristive behavior can be realized by variable voltage stimulus and illumination duration. The Al/MWCNT@GQDs/ITO memristor presented programmed and erased dual‐tunable performance with improved reproducibility. In the MWCNT@GQDs active layer, the GQDs could serve as a nanoscale oxygen reservoir, locally forming oxygen vacancies under voltage bias. The introduction of GQDs into the composite layer triggered band alignment and increased tunneling current, reduced power consumption, and improved device stability. Remarkably, the high‐sensitivity GQDs also demonstrated tremendous potential for developing stimulus‐responsive memristors. In comparison with the bulk carbon materials, the CDs‐based memristors based on CDs or their composite materials normally show excellent memristive performance, including high uniformity, stability, and narrow switch voltage distribution.^[^ ^61^ ^]^

### 5.2 CDs on Inorganic Substances

Later, a BT memristor with the structure of Ag/GQDs/TiO*_x_*/F‐doped SnO_2_ was fabricated by Zhou et al.^[^ ^62^ ^]^ As shown in Figure 5d, the negative photoconductance effect was found in HRS, which could be explained by the excitation, migration, and compensation of oxygen vacancy at the organic–inorganic interface. In this principle, the injected electron was efficiently restricted due to the variation in the charge distribution. Moreover, the simple logic operation was implemented by using a memristor point array. The metal electrodes in memristor devices also greatly impact their electrical behaviors. Some metals, such as Ag, Cu, and Al, have been widely utilized as active top electrodes. During the device operation, the metal electrode is capable of producing a redox reaction to form Ag^+^ and Cu^2+^, followed by the migration process of metallic ions. Therefore, the threshold voltage of the device may show a dispersion distribution, caused by the random diffusivity of metallic ions in memristors.^[^ ^63^ ^]^ After the introduction of CDs, the growth and nucleation position of CFs can be well controlled, leading to an obviously concentrated threshold voltage distribution and optimized memory performance. Intriguingly, the diffusion of metallic ions is analogous to the dynamic change of Ca^2+^ in biological synaptic cells.^[^ ^64^ ^]^ This result suggests that the CDs‐based memristor owns a great possibility to simulate bionic devices and human sensory perception systems.

## 6 Artificial Synapses and Neuromorphic Computing

To break von Neumann bottleneck, advanced computing algorithms and device architectures are proposed, being inspired by the human brain computing network. Specifically, the state‐of‐the‐art bionic neuromorphic system has been developed to realize high‐frequency data transmission, processing, and ultrahigh‐density information storage.^[^ ^65^ ^]^ In recent years, memristors with gradual change behaviors in current have shown the possibility of mimicking biological synaptic signals, and further implementing hardware operation in neuromorphic computing systems. Therefore, the memristor‐based artificial synapses with learning, memory, and computing functions have attracted much attention. Up to date, a diversity of advanced memristive materials have been developed, including traditional inorganic metal oxide semiconductors, perovskites, MXenes, black phosphorus, etc.^[^ ^66^ ^]^ Among them, 0D CDs exhibit some unique advantages for artificial synapse applications: 1) CDs with controllable nanometer scale show superior mechanical flexibility and transferability; 2) Compared to conventional organic functional materials, CDs and their derivatives have better thermal and chemical stability; 3) CDs own abundant oxygen‐bearing functional groups at surface, which may induce resistance switching performance through the migration of intrinsic oxygen vacancy; 4) CDs‐based memristors with optimized stability and endurance performance, show great potential in the field of artificial synapses and neuromorphic computing applications.

Over the past years, CDs combined with various organic molecules or 2D platforms have been widely applied in memristive devices, however, very few works emphasized CDs‐based artificial synapses (**Table** **2**). In 2017, Choi et al. found a pinched hysteresis in the device of Al/poly(3,4‐Ethylenedioxythiophene) Polystyrene Sulfonate (PEDOT:PSS)@GQDs/ITO with increased/decreased conductance under continuous positive/negative voltage sweeps, which could be further used for the fingerprint of synapses.^[^ ^67^ ^]^ The Yan group has also been dedicated to studying carbon‐based memristors and artificial synaptic devices.^[^ ^68^ ^]^ For instance, a memristor with the structure of Pd/CDs/Ga_2_O_3_/Pt was fabricated, and presented the learning rules of short‐term plasticity (STP), long‐term potentiation (LTP), long‐term depression (LTD), and spike‐timing‐dependent plasticity (STDP).^[^ ^3^ ^]^ A single perception layer (SPL) was constructed by a memristor‐based artificial neural network (ANN), which could realize Pavlovian associative learning and extinction behaviors, and digit recognition with an accuracy of 92.63%. Another Ag/Zr_0.5_Hf_0.5_O_2_/GQDs/Ag device also showed bidirectional progressive conductance tuning due to the synergistic effect of tunneling and electrochemical metallization.^[^ ^69^ ^]^ This device was also employed to mimic the synaptic functions of STDP, short‐term paired‐pulse facilitation (PPF), and nonlinear transmission properties. Besides, the introduction of GQDs into Zr_0.5_Hf_0.5_O_2_ can prevent the migration of Ag^+^ and enhance localized electrical field function in the device of Ag/Zr_0.5_Hf_0.5_O_2_/GQDs/Zr_0.5_Hf_0.5_O_2_/Pt, achieving the synaptic learning and memory functions of STP, LTP, STDP, and PPF.^[^ ^70^ ^]^

**Table 2:** The representative parameters of CDs‐based memristor and artificial synapses

Device structure	CDs material	Memory type	ON/OFF ratio	Retention time [s]	Synaptic function and application	Bending cycle or radius	Mechanism	Ref.
Al/PVP@NCDs/ITO	PVP@NCDs	A‐RS	—	—	EPSC, PPF, STDP, STP and LTP, ANNs	250 cycles, 5.2 mm	Charge trapping	[73]
Pd/CDs/Ga_2_O_3_/Pt	CDs	D‐RS	10^2^	4.5 × 10^4^	LTP, LTD, STDP, SPL	—	CCFs	[3]
Ag/Zr_0.5_Hf_0.5_O_2_/GQDs/Ag	GQDs	Adjustable conductance	<10	—	STDP, PPF	—	Ion migration	[69]
Ag/HfO_2_/GQDs/Pt	GQDs	D‐RS	10^6^	10^4^	LTP, EPSC, ANNs, numerical recognition	—	Ion migration	[76]
Ag/Zr_0.5_Hf_0.5_O_2_/GQDs/Zr_0.5_Hf_0.5_O_2_/Pt	GQDs	Adjustable conductance	10^2^	—	TP, LTP, STDP, and PPF	—	ECM	[70]
Al/PEDOT:PSS@GQDs/ITO	PEDOT:PSS@GQDs	Adjustable conductance	—	—	Pinched hysteresis, tactile sensor	—	Interface/surface traps	[67]
Ag/PMMA@CDs/FTO	PMMA@CDs	D‐RS	10^3^	10^4^	—	—	Charge trapping	[49]
Au/CDs@silk/ITO	CDs@silk	D‐RS (WORM)	5 × 10^3^	10^6^	—	—	ECM	[35]
AgNWs/CDs@PVP/AgNWs/PVP	CDs@PVP	D‐RS (DRAM)	10^5^	10^4^	—	100 cycles	Charge trapping	[57f]
AgNWs/CDs@PVP/AgNWs/Gelatin	CDs@PVP	D‐RS (Flash)	10^2^	10^4^	—	15 mm	Charge trapping	[19e]
TiN/CNBs/ITO/mica	CNBs	D‐RS (Flash)	10	4 × 10^4^	LTP, LTD, STP, biological vision	—	—	[78d]

**Figure 6:**
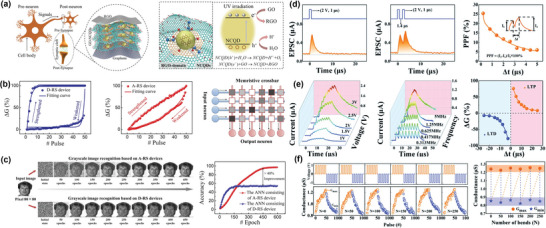
a) All‐carbon memristive synapse fabricated by a photo‐reduction method. b) Conductance potentiation/depression of the CDs‐based devices in D‐RS and A‐RS behaviors. c) Pattern recognition mimicked in the ANNs involving the D‐RS and A‐RS devices. Reproduced with permission.^[^ ^71^ ^]^ Copyright 2020, Springer Nature. d) Synaptic learning EPSC and PPF functions simulated in the Al/PVP@NCDs/ITO memristor. f) The pulse amplitude, frequency, and relative changes of the synaptic weight. Reproduced with permission.^[^ ^73^ ^]^ Copyright 2022, American Institute of Physics.

In 2020, Lin et al. prepared an all‐carbon artificial synapse, where the nanocomposite comprises the photoreduction of GO and nitrogen‐doped CDs (NCDs) through a one‐step reaction between C_3_N_4_ and ethane diamine.^[^ ^71^ ^]^ **Figure** **6**a depicts the BT structure of GO@NCDs‐based artificial synapse device. Given that both C_3_N_4_ and CDs possess high photocatalytic activity and photosensitivity, UV irradiation was employed on the composite film to generate more photogenerated carriers. As shown in Figure 6b, the reduced graphene oxide (RGO)/GO@NCDs/graphene structural devices showed D‐RS behavior at low NCDs concentrations (below 20 wt%), which is unsuitable for bio‐realistic emulation of synaptic adaptive functions. The analog‐type resistive switching (A‐RS) behavior only appeared at the moderate concentration of CDs by 30–60 wt%. Further synaptic functions of PPF, excitatory postsynaptic current (EPSC), and long‐term STDP were achieved to simulate the learning behavior of biological synapses. Significantly, the device could maintain high stability even under the harsh condition of 450 K. Compared with the D‐RS memristive behavior, the A‐RS‐type memristor has the potential applications of image recognition in complex neuromorphic computing systems.^[^ ^72^ ^]^ An ANN simulator was established by integrating 80 × 80 all‐carbon memristive cells in a crossbar array (Figure 6c). Every synapse cell could be analogous to different pixel dots. Based on this rule, the input grey image could be learned by A‐RS‐type ANN system with 96.7% accuracy, which was 40% higher than that of the D‐RS memristor system.

Recently, Zeng et al. reported a case of A‐RS behavior based on NCDs, where continuous resistance‐state variation could be implemented to simulate synaptic functions.^[^ ^73^ ^]^ In their work, citric acid and urea were respectively utilized as the carbon and nitrogen sources during the fabrication of CDs. The electron‐rich N groups provided more defects on the surface of NCDs, readily facilitating the generation and recombination of electron‐hole pairs. The device with Al/PVP@NCDs/ITO structure was fabricated in a crossbar array. As shown in Figure 6d, a single or two voltage pulses by 2 V were applied to the memristor, both showing temporary signal enhancement and further slow decay to their initial state. These electrical signals, namely EPSC function, are analogous to Ca^2+^ dynamics in biology. In addition, the post‐spike was strongly strengthened, however, the PPF index declined with increasing interval time, which could be calculated according to the following Equation (1).

(1) $$\mathrm{PPF}\;=\;\left(I_{2}\;-I_{1}\right)/I_{1}\;\times \;100\%$$

Moreover, an enhanced memory retention was obtained through increasing pulse amplitude, width, and frequency, implying that the possible transition from STP to LTP, and the typical STDP learning principle, took place in this memristor (Figure 6e). The synaptic weight (∆*W*) is one of the essential learning laws to evaluate the synaptic functions, which is expressed by the following two exponential fitting Equations (2) and (3). When the presynaptic spike precedes the postsynaptic spike (i.e., ∆*t* > 0) or falls behind the postsynaptic spike (i.e., ∆*t* > 0), ∆*W* should be respectively defined as LTP (∆*W* ^+^) and long‐term depression (LTD) (∆*W* ^―^).

(2) $$\Delta t\;>0,\;\Delta \;W^{+}=\;A^{+}\exp\left(-\;\Delta t/\tau_{+}\right)$$

(3) $$\Delta t<0,\;\Delta \;W^{-}=\;A^{-}\exp\left(\Delta t/\tau_{-}\right)$$

In these equations, *A* ^+^/*τ* ^+^ and *A* ^−^/*τ* ^−^ are the amplitude parameter and time constant for LTP and LTD, respectively. Thus, all the results showed that the plastic A‐RS behavior was superior to D‐RS performance in simulating synapses. Besides, the memristive devices exhibited no significant change in LTP and LTD process after bending several cycles (Figure 6f), manifesting viable flexibility against mechanical strain. Although CDs‐based artificial synapses and neuromorphic computing applications have entered researchers' vision, the current focus still lies in preparing novel CDs materials with clear geometry, composition, and structure. More investigations are required to explore the mechanism, integrated device manufacturing, etc.^[^ ^74^, ^75^ ^]^

## 7 Switching Mechanism of CDs‐Based Memristor and Synaptic Device

### 7.1 Ion Migration

Although the mechanism of CFs has been widely acknowledged for two‐terminal memristors, different arguments still exist in the formation and fracture of the nanoscale conducting channels. Some researchers found that the ion migration (IOM) between two electrodes driven by an external electrical field may trigger variable resistances.^[^ ^3c^ ^]^ Different metal anions (e.g., Ag^+^, Cu^2+^) and cations (e.g., oxygen, sulfur ions, and ammonium radical) have been explored over the past years, corresponding to the typical electrochemical metallization memory (ECM) and valence change memory (VCM) mechanisms. In CFs‐driven memristors, the uniformity of ion transport or diffusion plays a significant role in determining the growth and fracture of dendritic filaments. Normally, the dendritic CFs are disordered, leading to an unstable memory behavior and high‐power consumption. Li et al. tried to solve the challenge of wide fluctuations of switching parameters in PMMA‐based memristors by inserting CDs, rather than inorganic particles (e.g., Cu_2_ZnSnS_4_, ZnO, and SnO_2_).^[^ ^49^ ^]^ As a result, optimal memristive properties such as low set/reset voltages and excellent switching repeatability were obtained. According to the COMSOL simulation, these factors contributed memristive behaviors to the redox reaction and migration of Ag^+^ (Ag → Ag^+^ + e^‒^) in the memristor under an electroforming voltage (Figure 4c). The Ag^+^ migrates across the PMMA film could be further reduced and electro‐crystallized at the cathode surface (Ag^+^ + e^−^ →Ag). Therefore, the electro‐crystallization process and electrochemical dissolution realize the formation and rupture of Ag filament, leading to the transformation between HRS and LRS. More importantly, the incorporation of CDs in PMMA could act as trapping centers to capture the carriers injected from the electrode. Thus, a powerful local electric field nearby was produced in PMMA to induce the ordered growth and fracture of conductive filaments, affording improved memristor performances.

**Figure 7:**
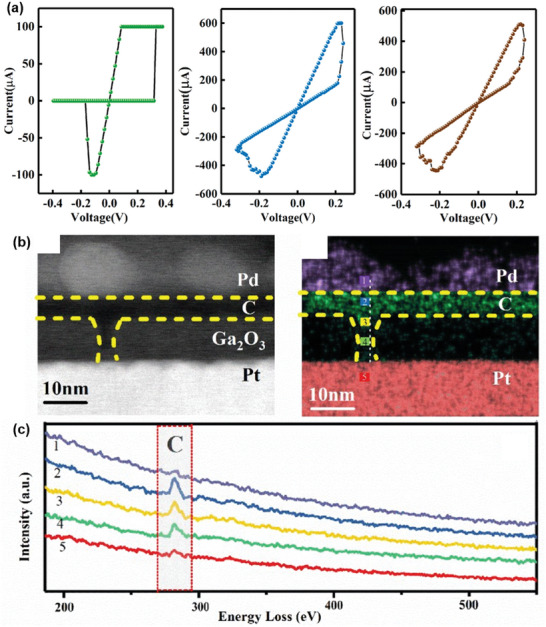
a) The *I*–*V* characteristics of the fabricated GQDs‐based memristor with low (left), moderate (middle), and high (right) densities. Reproduced with permission.^[^ ^70^ ^]^ Copyright 2019, American Institute of Physics. b) Transmission electron microscopy (TEM) picture and c) corresponding EELS mapping of the device at “ON” state. Reproduced with permission.^[^ ^73^ ^]^ Copyright 2020, Royal Society of Chemistry.

In CDs‐based synaptic devices, such as Ag/Zr_0.5_Hf_0.5_O_2_/GQDs/Ag, GQDs form a local electric field around Zr_0.5_Hf_0.5_O_2_ during the resistance switching, which induces the formation of Ag^+^ CFs in an orientation assignment.^[^ ^70^ ^]^ Besides, the densities of GQDs also have a great effect on the resistive switching behavior of Ag/Zr_0.5_Hf_0.5_O_2_/GQDs/Zr_0.5_Hf_0.5_O_2_/Pt memristors. At low concertation, the device showed D‐RS performance, however, the device conductance gradually changes at a certain density of GQDs (**Figure** **7**a). When the density of GQDs reached a high level, the memristor could mimic the synaptic learning and memory functions of STP, LTP, STDP, and PPF. The underlying mechanism might be that GQDs can prevent the migration of Ag^+^ and oxygen ions. However, with the assistance of localized electrical field function of GQDs, the device can form partial conduction/leakage through regulating the density of CDs in the device. Therefore, this result strongly suggests that the density of GQDs is a significant factor to modify the type of filament, leading to different device characteristics in artificial neuromorphic computing systems.

### 7.2 Charge Trapping/Detrapping

In most Ag anode‐based devices, ECM is the main mechanism for resistive transitions. However, some factors might change in Al, Au anode‐based devices. For example, Lv et al. found that the carrier transport behavior in Al/CDs‐silk/ITO and Au/CDs‐silk/ITO devices obeys the space‐charge limited conduction (SCLC) mechanism.^[^ ^35^ ^]^ Because of the non‐uniform particle surface sites of CDs, silk and the interface of CDs‐silk/electrode, a lot of trap sites (e.g., oxygen vacancy) exist in insulating CDs‐silk medium. Besides, the higher first‐ionization potential in these devices restrains the injection and migration of metal cations. First, the injected charges from the electrode will fill the charge traps in active CDs‐silk layers under a voltage bias. In this stage, the density of the generated free carriers is larger than that of injected charge carriers, following the Ohmic conduction mechanism. With the voltage increasing, these encapsulated charge carriers in trap sites will form a conductive path in the active layers, which is dominated by SCLC transport and Child's law. When the concentration of injected carriers is higher than that in an equilibrium state, all the trap sites are filled by the abundant charge carriers, resulting in the aggregation of charge carriers at the electrode interface.^[^ ^4c^ ^]^

Similarly, Yan et al. clearly figured out the conduction mechanisms of CDs‐based artificial synaptic device (Pd/CDs/Ga_2_O_3_/Pt) using the *I*–*V* fitting curve and TEM measurements.^[^ ^3a^ ^]^ The cross‐sectional TEM images presented the microstructure of the device in the “ON” state, where CDs insertion could be well observed (Figure 7b). According to the electron energy loss spectroscopy (EELS) mapping, CCFs was formed between the top and bottom electrodes in Ga_2_O_3_ films, which was consistent with *I*–*V* fitting results of the Ohmic conduction mechanism (Figure 7c). The formation and rupture of CCFs were also verified through model investigation. The CCFs was simulated as a one‐dimensional nanowire with a length of 10 nm on a heated table (300 K). The linear relationship between temperature coefficient and resistance could also support the CCFs mechanism. Furthermore, the electric‐field lines converged around the CDs, leading to an enhanced electric field, and the nucleation probability. After the formation of carbon nucleus, the electrochemical deposition rate would be increased under an enhanced electric field. Thus, the emulation of STP, LTP, LTD, STDP learning and Pavlovian associative learning rules could be simulated in the CDs‐based synaptic devices.

### 7.3 Phototunable Effects

To endow more possibility to remotely control memristors with nearly arbitrary spatial and temporal precision, a variety of noninvasive stimulus can be applied, for instance, external photic and magnetic stimulations.^[^ ^3d^ ^]^ Considering that CDs can act as electron acceptors or donators under light irradiation, the charge trapping capacity of CDs is capable of changing under light irradiation. Lv et al. investigated the photogate effect by in situ KPFM and SEM/EDS microanalysis. Under UV light illumination, the device performs an apparent decreasing trend on switching voltage and current, which may offer advantages of developing low‐power memristor devices (Figure 4e).^[^ ^35^ ^]^ According to the experimental results, the active layer owns a stronger electron‐trapping ability (potential: 0.09 V) than that of holes (potential: 0.03 V) after incorporating CDs, implying that electrons were trapped in a deeper level than holes. In addition, the potential change of the un‐injected area displays decreased value from 1.30 to 0.62 V, while the potential value of the electron‐injected area increased from 0.09 to 0.15 V. However, the potential value of hole‐injected areas showed negligible variation even after UV light illumination. Thus, the charge‐trapping capability is largely enhanced in CDs‐based active medium. Under UV irradiation, more electron‐hole pairs can be produced from CDs via absorbing high‐energy photons. The excitons are divided into electrons and holes between CDs and composite component, where the photogenerated electrons could be captured by CDs and the interface. Based on ion migration and charge trapping/detrapping mechanism, the photogate effect can be accelerated to realize phototunable memristive behavior and prominently enhanced operation speed and control capability.

## 8 Memristor+ (Future Prospects)

Compared to black carbon materials, fluorescent CDs astonishingly shine with excellent luminescent properties and high emission efficiency. To date, various photoinduced applications of CDs have been explored, such as bioimaging, optical sensing, and pesticide monitoring. However, the development of photoinduced electronic devices still lag behind the optical emission application. Thus, developing the combination strategy of photo‐ and electro‐response of CDs materials is conducive to promoting more advances in multi‐dimensional applications. To figure out the optoelectronic characteristics of CDs in depth, various modern molecular models should be established from different levels of electrons, atoms, and molecules, which have to correlate tightly with the experimental results.^[^ ^77^ ^]^

### 8.1 Controllable Morphology and Nanostructure of CDs

**Figure 8:**
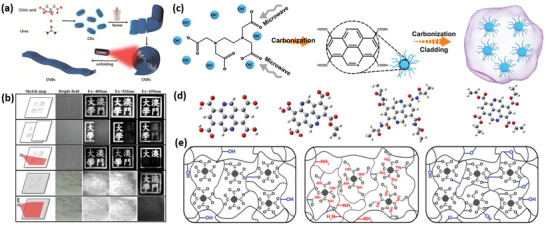
a) Schematic illustration of structural evolution from CDs to CNRs and CNBs. b) Encrypted patterns and logic operation utilizing CNR and CNB solutions as inks on a paper. c) Schematic diagram of the synthesis of cross‐linking CDs. Reproduced with permission.^[^ ^6a^ ^]^ Copyright 2021, American Chemical Society. d) The model molecular structure of CDs with carboxyl group sites, and further attaching polymer fragments. e) The network structure of CDs@TEOS, CD@PAM (N), and CD@PVA by cross‐linking. Reproduced with permission.^[^ ^80^ ^]^ Copyright 2022, Wiley.

For carbon‐based memristive materials, the intrinsic crystal structure and packing orientation significantly influence the electrical behavior of RRAM.^[^ ^78^ ^]^ Thus, establishing a clear structure–property relationship at molecular accuracy becomes one of the most important topics. Zhang et al. previously reported that the celestine blue (CB) dye molecules could both display Flash‐ or WORM‐type (write once read many times) memory performances.^[^ ^2c^ ^]^ Different nanostructures of nanopillars (NPs) and nanofibers (NFs) were assembled through the spin‐coating method and electrophoretic‐induced self‐assembly deposition (EPAD) technique, respectively. As a result, the NFs‐based memristor presented Flash‐type memory behavior, whereas NPs‐based device showed WORM‐type performance. Similarly, most CDs derivatives can be obtained through tuning size, controlling morphology, and adopting N‐doping strategy, which deserves to be used in memristors. A family of CDs derivatives with different nanostructures and morphologies, such as carbon nanobelts (CNBs), carbon nanorolls (CNRs), etc., have been well investigated, which holds the promise for memristive application. These novel carbon nanostructures are constructed by noncovalent bonds (e.g., H‐bonding or van der Waals effect), which are basically distinct from some traditional carbon nanomaterials (e.g., CNTs, fullerenes, and graphene) stabilized by covalent bonds. Recently, Liang et al. reported a solvothermal fusion method to form a CNRs assembly through a dehydration procedure of their surface functional groups (i.e., carboxyl, amino, carboxylate).^[^ ^6a^ ^]^ As illustrated in **Figure** **8**a, the CNRs could gradually unwind to CNBs under laser irradiation, which can be explained by the decreased surface groups and weakened intermolecular forces. The transformation from CDs to CNRs and CNBs brings unique optical absorption and emission spectra. Notably, the properties of photothermally induced emission changes can be utilized to construct multilevel information storage and encryption. The image stamped with CNRs ink showed excitation‐dependent emissions under the irradiation of 405 nm and 532 nm, displaying the logo of the University of Macau in Chinese characters (Figure 8b). However, the information emission disappeared under 655 nm excitation, due to the transition from CNRs to CNBs. These CDs derivatives with different shapes and morphologies indicated peculiar information‐storage characteristics, offering more opportunities to develop synergistically photoelectric memristors.

In addition to CNRs and CNBs, CDs also have some other derivatives with different structures and components. During the synthetic process, various supramolecular interactions, such as H‐bonding, amphiphilic interactions, *π*–*π* interactions, electrostatic, and van der Waals forces, can form non‐covalent or covalent assembly of CDs in collaboration, named as supra‐CDs. For instance, it was reported that hydrothermal cross‐linking polymerization may happen dehydration reactions during the synthesis of CDs.^[^ ^79^ ^]^ As shown in Figure 8c, Wang et al. used ethylenediaminetetraacetic acid tetrasodium salt (EDTA·4Na) to implement intersystem crossing (ISC) processes through a microwave method.^[^ ^80^ ^]^ Then, PVA, polyacrylamide (PAM), or tetraethyl orthosilicate (TEOS) were coated outside of CDs to build core–shell structure and gain enhanced ISC in the following microwave step (Figure 8d). The introduced polymers containing a number of functional groups and side chains, such as hydroxyl, ethoxy, and amino, generated more cross‐linking polymeric structures (Figure 8e). On the one hand, this synthetic procedure could provide more abundant energy levels, facilitating efficient charge‐carrier transfer between the CDs and shells. On the other hand, it improves spin‐orbit coupling by optimal singlet‐to‐triplet excited states in the CDs, which passivates the non‐radiative decay of the excited triplet state.^[^ ^81^ ^]^ Recently, Ai et al. produced self‐assembled 1D CNBs through the reaction between 0D CDs and aromatic ligands. The self‐assemblies were utilized as the active component in a memristor device, showing a much more stable data storage capacity prolonging to one month. Besides, the device could also be applied to simulate a biological vision system and rapidly identify Chinese characters.^[^ ^78^ ^]^ We conclude that the cross‐linked polymeric CDs and core‐shell structured CDs exhibit unique electron‐transfer properties, hence, they would become a more appealing candidate for memristor and other electronic applications.

### 8.2 Photosensitive Logic Sensors

**Figure 9:**
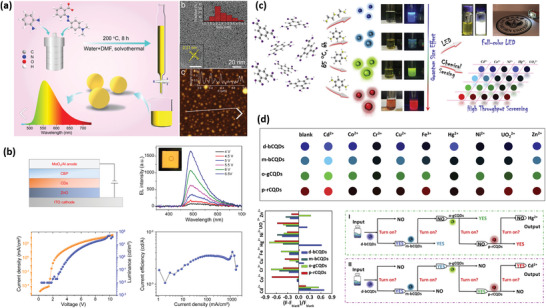
a) Schematic illustration of the synthesis process of NCDs, and the morphology of CDs. b) Structure and properties of the CD‐based electroluminescent device. Reproduced with permission.^[^ ^84^ ^]^ Copyright 2022, Wiley. c) The synthetic procedures of CDs and their application in logic chemosensing. d) The CDs‐based logic gates for recognizing Hg^2+^(I) and Cd^2+^(II). Reproduced with permission.^[^ ^85^ ^]^ Copyright 2022, Elsevier.

Besides the electrical stimulus, the memristor and artificial synapses can also be affected by other external stimuli, such as pressure, photo, and magnetic effect. Zhang et al. demonstrated a kind of hetero‐bilayer films of perylene (Py) and GO prepared by photosensitive interfacial co‐assembly. The Py/GO‐based memristive device not only showed ultrahigh specific light detectivity, but also owned excellent photonic synaptic behaviors with a PPF index of 214% in neuroplasticity. Inspired by this kind of work, developing stimuli‐responsive memristive materials has drawn much attention.^[^ ^82^ ^]^ To our knowledge, CDs materials have been explored for high‐performance solid‐state lighting devices, demonstrating the feature of environmental friendliness, high efficiency, and long lifetime. Analogous to constructing localized energy states and confinement potentials in some traditional SQDs via heteroatom doping, Lou et al. attempted to immobilize the photoinduced electron/hole pairs to the radiative centers, aiming to realize high‐efficiency localized excitons.^[^ ^83^ ^]^ As shown in **Figure** **9**a, pyrrolic nitrogen was chosen as N source precursors, forming bound excitons and the symmetry break of the *π*‐electron conjugation. In addition, the electroluminescent device with a low threshold voltage of 3.0 V was fabricated (Figure 9b), indicating a low‐charge injection barrier under the external electric field.^[^ ^84^ ^]^ This result becomes robust evidence to infer that a heteroatom‐doped CDs layer may also be employed for electric‐induced memristive devices. Tong et al. developed a mild condensation strategy to fabricate CDs products. In the solvents of diethylenetriamine (DETA), m‐phenylenediamine (m‐PDA), o‐phenylenediamine (o‐PDA), or p‐phenylenediamine (p‐PDA), 1,3,5‐benzenetricarboxylic acid (BTCA) was added as the sp^2^ carbon source (Figure 9c). Under a mild temperature of 85 °C and a nearly atmospheric pressure (≈1.88 bar), the CDs could be rapidly synthesized through a one‐pot method.^[^ ^84^ ^]^ As seen in Figure 9d, full‐color fluorescent emission was obtained according to the as‐prepared solid‐state CDs. The fluorescence properties could also be regulated by changing the chemical environment during synthesis process, such as pH and metal ions, which can be explained by the possible protonation/deprotonation, and coordination reaction among abundant active sites. Consequently, the photosensitive CDs device offered a valuable chance to identify various metal ions, and form high‐throughput logic gate sensors for multiple applications (e.g., gas test, food analysis, and environmental analysis). It is logical to suppose that the combination of memristor and photosensitive sensor will generate a more prosperous and deeper logic integration to detect complex systems in a precise way.

### 8.3 Nanomemory Cells

**Figure 10:**
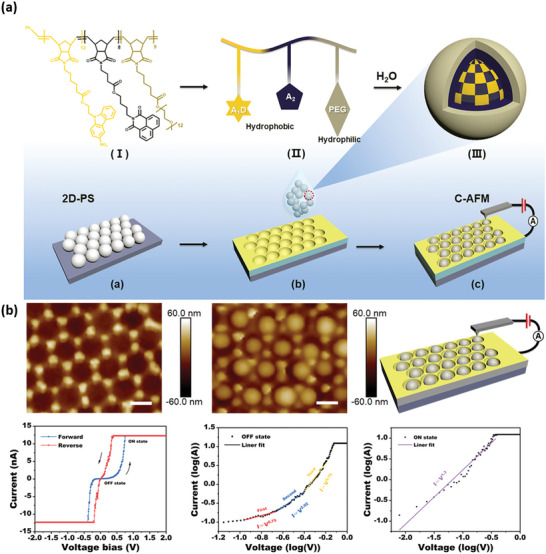
a) Schematic diagram of the self‐assembly of the amphiphilic triblock copolymer and fabrication of the nano‐memory device. b) The C‐AFM measurements and corresponding electrical performance. Reproduced with permission.^[^ ^2b^ ^]^ Copyright 2022, Wiley.

As mentioned above, downscaling the sizes of memory cells is one of the main limitations of developing ultrahigh‐capacity data storage technology. The current commercial data storage density is about 10^8^ bits cm^−2^, lagging far behind the expected demand of 10^12^ bits cm^−2^. To increase the density of device cells per unit area, some technical strategies have been proposed, such as the integration of crossbar arrays, the lithography technique, the anodic aluminum oxide patterning technique, and so on. Among them, the nanoparticle assembly technique has unique superiority in fabricating nano‐memory cells. Recently, Wang et al. synthesized a triblock amphiphilic copolymer by ring‐opening metathesis polymerization (ROMP) among the carbazole and naphthalimide monomers.^[^ ^2b^ ^]^ By adjusting assembly conditions, the N, O‐doped carbon materials can self‐assemble into nanospheres with an average size of 80 nm (**Figure** **10**a). Further efforts were made to disperse these nanospheres on a polystyrene (PS) substrate to form nano‐memory cells in an array. According to the conductive atomic force microscopy (C‐AFM) measurements, each nano‐memory cell displayed predominant D‐RS behavior under voltage stimulus (Figure 10b). Analogous to the polymeric nano‐assembly, the nanoscale CDs also hold great promise for preparing nano‐memory cells and array matrix, which can be further developed for high‐density data storage and logic operation.

### 8.4 Integrated Tactile Sensory System

**Figure 11:**
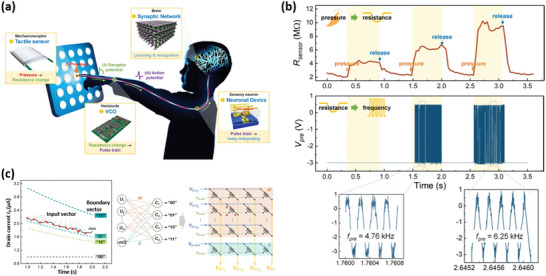
a) Conceptual graph of the tactile sensor system analogous to a biological sensor system. b) The integration of tactile sensor devices with the VCO circuit. c) The learning and recognition principle in the carbon‐based transistor array. Reproduced with permission.^[^ ^89^ ^]^ Copyright 2020, Springer Nature.

Integrating memristors with some traditional circuits has become one kind of new research tendency. Recently, the Yan group utilized the established Ag/HfO_2_/GQDs/Pt memristor and STM32 microcontroller chip controlling to construct a low‐, high‐, and band‐pass circuit‐based filter.^[^ ^76^ ^]^ By changing the output signal that was controlled by memristive resistance, the circuit‐based filter is capable of adjusting the cutoff frequency. More intriguingly, the memristor‐simulated artificial synapses and neurons exhibited the ability to realize image cognitive tasks in crossbar arrays. To our knowledge, most previous works only focused on the emulation of time‐dependent input stimuli signal in the leaky integrate‐and‐fire (I&F) neuron model. To further promote the development of artificial synapses and neuromorphic computing, some recent studies integrated spatial (e.g., tactile, visual, or pressure stimuli) or temporal signals to establish the human sensory perception system.^[^ ^86^ ^]^ For example, Li et al. developed an alcohol gas recognition system by integrating COFs‐based sensory memristor and the *k*‐nearest neighbors (KNN) classification algorithm. The accuracy of alcohol recognition can reach up to 87.2%.^[^ ^87^ ^]^ Wang et al. integrated the lobule giant movement detector (LGMD) visual neuron into a light‐mediated memristor with Ag/black phosphorous@perovskite quantum dot/ITO structure, forming a wide field‐of‐view (FoV) detection capability and obstacle avoidance response.^[^ ^88^ ^]^ As shown in **Figure** **11**a, Kim et al. designed a biorealistic tactile sensor system, composing sensory neurons and perceptual synaptic networks. In detail, the sensory neuron functions in human body can be respectively simulated by a tactile sensor, a voltage‐regulation oscillator circuit, and one neuronal transistor array.^[^ ^89^ ^]^ Figure 11b shows the photograph of the tactile sensor device and voltage‐regulation oscillator circuit. It was found that the resistance changes as the pressure increases, subsequently transforming the frequency of the presynaptic spike in neuronal devices. Four boundary vectors were defined as “00,” “01,” “10,” and “11,” to describe the tactile patterns. Here, this semivolatile CNT transistor is capable of switching the operation mode under the voltage bias. It means that the semivolatile CNT synaptic transistor can realize both neuronal and synaptic functions (Figure 11c). In this regard, the artificial perceptual sensory system could mimic the human biological sensory system by receiving transformed stimuli signals. Till now, there has been no report about the CDs‐based memristor for the integrated tactile sensory system. In view of the development of CDs materials, we envision bright prospects to establish more efficient integrated devices and sensory systems.^[^ ^90^ ^]^

## 9 Conclusions

CDs and their derivative composites have been successfully explored in memristor, artificial synapse, and neuromorphic computing applications, due to their excellent properties of biocompatibility, low toxicity, environmental friendliness, and simple preparation. As compared with traditional organic carbon materials, CDs‐based electronic devices are more resistant to temperature, but more sensitive to heat and light. This review summarizes recent advances in the synthetic route of diversified CDs and their derivatives, switching mechanisms, smart memristors, artificial synapses, and neuromorphic computing applications. Despite these achievements in recent years, there remain a number of challenges and limitations: 1) The introduction of CDs into self‐assembled films helps to improve device stability and reproducibility. However, it should be noted that CDs have a lot of active groups (such as amides, hydroxyls, or carboxyls) anchored onto their surface, which may not be conducive to long‐term stability under ambient conditions. Thus, developing a surface passivation strategy to prepare desired CDs candidates could assure solid memristive performances. 2) Normally, most CDs exhibit low quantum yields below 10%, making them unsuitable for multifunctional photosensitive memristors, artificial synapses and neuromorphic applications. How to prepare CDs materials with a high quantum yield is one of the popular research topics. 3) Given that nanoscale CDs often serve as memristive materials, the integration of nano‐memory cells with nanostructured assembly to fabricate high‐density nanoarrays is fascinating but challenging. 4) The investigation of CDs‐based artificial synapse is still in its infancy, despite the excellent structural stability, nano‐size, and quantum confinement effects of CDs. Novel bionic synaptic devices with exceptional transparency, mechanical flexibility, and transferability need to be pursued. 5) An integrated device with the integration of sensory storage and computation functions should be constructed for future human sensory perception systems. Furthermore, advanced algorithms and logic operations are urgently needed for competitive neuromorphic platforms. In general, we anticipate that this review will lead to further in‐depth research on CDs‐based smart memristors, low‐power artificial synapses, and neuromorphic computing applications.

## Conflict of Interest

The authors declare no conflict of interest.
